# Complete Genome Sequence of *Staphylococcus pseudintermedius* Type Strain LMG 22219

**DOI:** 10.1128/genomeA.01651-16

**Published:** 2017-02-16

**Authors:** Mohamed A. Abouelkhair, Matthew C. Riley, David A. Bemis, Stephen A. Kania

**Affiliations:** a Department of Biomedical and Diagnostic Sciences, Knoxville, Tennessee, USA; b Faculty of Veterinary Medicine, University of Sadat City, Menoufia, Egypt

## Abstract

We report the first complete genome sequence of LMG 22219 (=ON 86^T^ = CCUG 49543^T^), the *Staphylococcus pseudintermedius* type strain isolated from feline lung tissue. This sequence information will facilitate phylogenetic comparisons of staphylococcal species and other bacteria at the genome level.

## GENOME ANNOUNCEMENT

Here, we present the first complete genome sequence of LMG 22219 (=ON 86^T^ = CCUG 49543^T^), the *Staphylococcus pseudintermedius* type strain isolated from lung tissue of a cat (1). *S. pseudintermedius* is a Gram-positive opportunistic bacterial pathogen (2) commonly associated with canine pyoderma and occasionally isolated from human infections (3–6). It was first defined as a unique species by Devriese et al. (1). This species is classified as a member of the *Staphylococcus intermedius* group, which also includes *S. intermedius* and *S. delphini*, as defined by Takahashi et al. (7). *S. pseudintermedius* is characterized by nonpigmented colonies surrounded by double-zone hemolysis on Columbia sheep blood agar. It is catalase-positive and coagulates rabbit plasma but is clumping-factor-negative in slide coagulase testing. *S. pseudintermedius* is positive for DNase, β-glucosidase, arginine dihydrolase, urease, nitrate reduction, pyrrolidonyl arylamidase, and ONPG (β-galactosidase). It does not produce β-glucuronidase and is susceptible to 8 μg ml^−1^ acriflavine and to novobiocin. It is also resistant to deferoxamine (1).

Whereas assays exist to differentiate *S. pseudintermedius* from other *Staphylococcus intermedius* group species, it may be misidentified in veterinary and human medicine (6, 8). Clinical treatment of infections relies upon accurate pathogen identification (9) and the availability of the complete genome for this organism may allow for genetic validation of phenotypic testing, as well as identification of new genetic targets for interspecies comparisons. Only genome sequences of *S. intermedius* and *S. delphini* (10) type strains are currently available, and consequently the elucidation of the genome sequence of the *S. pseudintermedius* type strain LMG 22219 will facilitate comparisons of different species and strains based on genetic analysis.

DNA was extracted and a library prepared using the Nextera XT library preparation kit in accordance with the manufacturer’s protocol. Sequencing was performed using Illumina MiSeq version 2 (Illumina Inc., USA), and *de novo* assembly was performed using Geneious version 9.1.6 (11). Automated annotation of the assembled contigs was performed using the NCBI Prokaryote Genome Annotation Pipeline (http://www.ncbi.nlm.nih.gov/genome/annotation_prok).

A total of 1,432,416 MiSeq paired-end reads were used to generate 50 contigs with >100× average depth of coverage. The LMG 22219 genome comprises 2,523,112 bp with 37.6% GC content, 2,242 predicted coding sequences, and 81 RNAs.

### Accession number(s).

This whole-genome sequence has been deposited at DDBJ/ENA/GenBank under the accession number MLGE00000000. The version described in this paper is the first version, MLGE00000000.1.

## References

[1] Devriese LA, Vancanneyt M, Baele M, Vaneechoutte M, De Graef E, Snauwaert C, Cleenwerck I, Dawyndt P, Swings J, Decostere A, Haesebrouck F 2005 *Staphylococcus* *pseudintermedius* sp. nov., a coagulase positive species from animals. Int J Syst Evol Microbiol 55:1569–1573. doi:10.1099/ijs.0.63413-0. 16014483

[2] Van Hoovels L, Vankeerberghen A, Boel A, Van Vaerenbergh K, De Beenhouwer H 2006 First case of Staphylococcus pseudintermedius infection in a human. J Clin Microbiol 44:4609–4612. 17050817 10.1128/JCM.01308-06 PMC1698428

[3] Börjesson S, Gómez-Sanz E, Ekström K, Torres C, Grönlund U 2015 *Staphylococcus* *pseudintermedius* can be misdiagnosed as *Staphylococcus aureus* in humans with dog bite wounds. Eur J Clin Microbiol Infect Dis 34:839–844. doi:10.1007/s10096-014-2300-y. 25532507

[4] Frank LA, Kania SA, Hnilica KA, Wilkes RP, Bemis DA 2003 Isolation of *Staphylococcus* *schleiferi* from dogs with pyoderma. J Am Vet Med Assoc 222:451–454. doi:10.2460/javma.2003.222.451. 12597417

[5] Lee J, Murray A, Bendall R, Gaze W, Zhang L, Vos M 2015 Improved detection of *Staphylococcus intermedius* group in a routine diagnostic laboratory. J Clin Microbiol 53:961–963. doi:10.1128/JCM.02474-14. 25502532 PMC4390641

[6] Sasaki T, Kikuchi K, Tanaka Y, Takahashi N, Kamata S, Hiramatsu K 2007 Reclassification of phenotypically identified *Staphylococcus intermedius* strains. J Clin Microbiol 45:2770–2778. doi:10.1128/JCM.00360-07. 17596353 PMC2045239

[7] Takahashi T, Satoh I, Kikuchi N 1999 Phylogenetic relationships of 38 taxa of the genus *Staphylococcus* based on 16S rRNA gene sequence analysis. Int J Syst Bacteriol 49:725–728. doi:10.1099/00207713-49-2-725. 10319495

[8] Weese JS 2013 Phenotypic identification of *Staphylococcus intermedius* may be inaccurate. J Clin Microbiol 51:734. doi:10.1128/JCM.02736-12. 23355726 PMC3553881

[9] Starlander G, Börjesson S, Grönlund-Andersson U, Tellgren-Roth C, Melhus A 2014 Cluster of infections caused by methicillin-resistant *Staphylococcus* *pseudintermedius* in humans in a tertiary hospital. J Clin Microbiol 52:3118–3120. doi:10.1128/JCM.00703-14. 24871217 PMC4136194

[10] Ben Zakour NL, Beatson SA, van den Broek AHM, Thoday KL, Fitzgerald JR 2012 Comparative genomics of the *Staphylococcus intermedius* group of animal pathogens. Front Cell Infect Microbiol 2:44. doi:10.3389/fcimb.2012.00044. 22919635 PMC3417630

[11] Kearse M, Moir R, Wilson A, Stones-Havas S, Cheung M, Sturrock S, Buxton S, Cooper A, Markowitz S, Duran C, Thierer T, Ashton B, Meintjes P, Drummond A 2012 Geneious basic: an integrated and extendable desktop software platform for the organization and analysis of sequence data. Bioinformatics 28:1647–1649. doi:10.1093/bioinformatics/bts199. 22543367 PMC3371832

